# Fetal growth pattern is regulated sex-specific dependent on maternal BMI

**DOI:** 10.1186/2194-7791-1-S1-A5

**Published:** 2014-09-11

**Authors:** B Brune, D Naehter, T Brune

**Affiliations:** University Children's Hospital Magdeburg, Magdeburg, Germany

## Aims

In previous studies we could show that male neonates with a Body Mass Index (BMI) <10P have a double risk to develop obesity during the first 6 years of life compared to females. As the main factor of influence for developing obesity we could identify the maternal BMI for both male and female infants. This leads to the hypothesis that maternal overweight has a sex specific influence on the intrauterine fetal growth pattern (Brune et al: Obesity 2010, 4:798-802).

## Methods

202 full-term infants (101 ♂/ 101 ♀) and 206 premature infants (109 ♂/ 97 ♀) were included into the study. To compare the data, birth weight and length of the full-term infants were adjusted to 36 GA (gestational age) and of premature infants to 30 GA using Prader Percentiles. After delivery, the premature infants received a standard nutrition and 6 weeks later weight and length were measured again. For all children the Ponderal Indices (PI) were calculated. Univariate covariance analysis was performed using PI as target size, sex or GA as fixed factors and maternal BMI before pregnancy as a covariate. We investigated whether there was a difference between interactions of PI depending on maternal BMI and children´s sex in full-term infants at the time of 36 GA and in premature infants both at 30 GA and at 36 GA.

## Results

The PI of female full-term infants correlated positively, that of male negatively with maternal BMI. There was no sex difference between premature infants at the time of delivery (p=0.2), but after receiving a standard nutrition during 6 weeks the correlation of PI with maternal BMI appeared significantly vice versa compared to full-term infants (p=0.01).

## Conclusion

Our results show that maternal overweight influences intrauterine growth pattern and therefore fetal programming sex-specific during the last 6 weeks of gestation.

